# Understanding the roles of the thylakoid lumen in photosynthesis regulation

**DOI:** 10.3389/fpls.2013.00434

**Published:** 2013-10-31

**Authors:** Sari Järvi, Peter J. Gollan, Eva-Mari Aro

**Affiliations:** Molecular Plant Biology, Department of Biochemistry, University of TurkuTurku, Finland

**Keywords:** NAD(P)H dehydrogenase, photosystem, proteome, thioredoxin, thylakoid lumen

## Abstract

It has been known for a long time that the thylakoid lumen provides the environment for oxygen evolution, plastocyanin-mediated electron transfer, and photoprotection. More recently lumenal proteins have been revealed to play roles in numerous processes, most often linked with regulating thylakoid biogenesis and the activity and turnover of photosynthetic protein complexes, especially the photosystem II and NAD(P)H dehydrogenase-like complexes. Still, the functions of the majority of lumenal proteins in *Arabidopsis thaliana* are unknown. Interestingly, while the thylakoid lumen proteome of at least 80 proteins contains several large protein families, individual members of many protein families have highly divergent roles. This is indicative of evolutionary pressure leading to neofunctionalization of lumenal proteins, emphasizing the important role of the thylakoid lumen for photosynthetic electron transfer and ultimately for plant fitness. Furthermore, the involvement of anterograde and retrograde signaling networks that regulate the expression and activity of lumen proteins is increasingly pertinent. Recent studies have also highlighted the importance of thiol/disulfide modulation in controlling the functions of many lumenal proteins and photosynthetic regulation pathways.

## INTRODUCTION

Photosystem (PS)I, PSII, and the light harvesting complexes (LHCI and LHCII), in concert with the cytochrome (cyt) *b_6_*f, ATP synthase, and the NAD(P)H dehydrogenase-like (NDH) are responsible for light harvesting and transduction of solar energy into chemical energy via photosynthetic electron transport (PET). These multi-subunit pigment–protein complexes are embedded in the highly folded thylakoid membrane, which encloses a continuous internal compartment known as the thylakoid lumen. The linear electron transport (LET) chain represents the predominant pathway of PET. Three major thylakoid membrane protein complexes – PSII, cyt *b_6_*f, and PSI – cooperate in LET in order to transport electrons from water molecules to oxidized nicotinamide adenine dinucleotide phosphate (NADP^+^). Photosynthetic water-splitting occurs at the lumenal side of PSII at the oxygen-evolving complex (OEC). Hydrogen ions accumulating in the lumen as a result of water-splitting and cyt *b_6_*f activity generate the proton motive force (*pmf*) that drives ATP synthesis. Lumenal proton concentration is also an important regulator of PET, triggering non-photochemical quenching (NPQ) of harvested energy and slowing down electron transfer in the cyt *b_6_*f complex under acidic lumenal conditions. While LET generates both NADPH and ATP, cyclic electron transport (CET) around PSI produces *pmf* and thus ATP without reducing NADP^+^ (Heber and Walker, 1992). To that end, the main role of PSI CET is to balance the production of ATP and NADPH according to metabolic needs and to alleviate stromal over-reduction (Shikanai, 2007).

**FIGURE 1:**
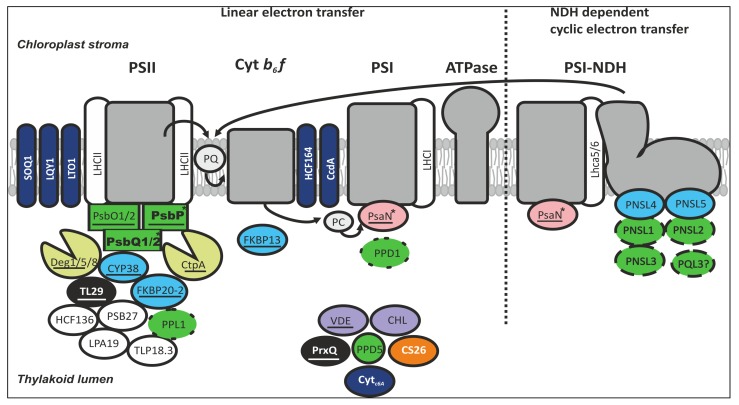
**The majority of thylakoid lumen proteins with experimentally verified roles are involved in the function of either the PSII complex or the PSI–NDH supercomplex.** The most abundant protein families in the thylakoid lumen are the OEC and OEC-like proteins (green), the immunophilins (blue), and proteases (yellow). In addition, the lumen proteome comprises peroxidases (black), photoprotective enzymes (purple), and several auxiliary proteins. The OEC proteins are proposed to function in water oxidation (square boxes), granal stacking (bolded), photosystem assembly (dotted outline), strigolactone biosynthesis (circle box), and NDH-dependent cyclic electron transfer (dashed outline). A high proportion of lumen proteins are thioredoxin targets (underlined). Regulation of thylakoid redox reactions involves membrane-embedded and soluble proteins (dark blue), and other lumen proteins are also implicated (white typeface). Lumen proteins with phosphorylation sites (asterisked) may be regulated by TLP18.3 phosphatase. Based on current knowledge, verified components of lumenal NDH subcomplex are not under post-translational regulation. No characterized lumenal proteins have so far been linked to the function of ATP synthase.

Although the photosynthetic apparatus and light-driven electron transport have been studied extensively, there remains a great deal to learn about factors that regulate PET according to the energy requirements of metabolic pathways and environmental cues. Recent characterizations of thylakoid lumen proteomes and analyses of the component proteins have revealed a range of novel proteins and protein families. Furthermore, the details of recent studies show that the lumen holds key factors for regulation and repair of the photosynthetic membrane, facilitating PET flexibility that is vital for efficient energy conversion. Here we review the current understanding of the functions of thylakoid lumen proteins in LET, CET, and PSII repair, and explore factors that regulate their expression, translocation, and activity (**Figure 1**; **Table 1**). Although uncharacterized lumen proteins have mainly been excluded from this review, their roles in PET regulation, retrograde signaling and/or acclimation are also likely to be vital for plant growth and development.

**Table 1:** Summary of characterized thylakoid lumen proteins in *Arabidopsis*.

Name	Gene code	Protein family/domain	Function/pathway	Signal peptide	Network	TRX target	Phosphorylation site	Reference
**PSII**								
PsbO1	At5g66570	OEC	Subunit of PSII OEC	Sec	C	Yes	–	Murakami et al. (2005)
PsbO2	At3g50820	OEC	Subunit of PSII OEC	Sec	C	Yes	–	Murakami et al. (2005)
PsbP1	At1g06680	OEC	Subunit of PSII OEC	Tat	C	Yes	Yes	Yi et al. (2007), Ifuku et al. (2008)
PsbQ1	At4g21280	OEC	Subunit of PSII OEC	Tat	C	–	–	Yi et al. (2006)
PsbQ2	At4g05180	OEC	Subunit of PSII OEC	Tat	C	–	Yes	Yi et al. (2006)
PSB27	At1g03600	PSB27	D1 processing	Tat	C	–	–	Chen et al. (2006)
LPA19	At1g05385	PSB27	D1 processing	Tat	–	–	–	Wei et al. (2010)
TLP18.3	At1g54780	Acid phosphatase	Degradation of D1, dimerization of PSII	Sec	C	–	–	Sirpio et al. (2007), Wu et al. (2011)
TL29/APX4	At4g09010	Peroxidase-like	Associated with PSII	Tat	C	Yes	–	Granlund et al. (2009b), Lundberg et al. (2011)
HCF136	At5g23120	–	Assembly of PSII	Tat	R	–	–	Meurer et al. (1998), Plucken et al. (2002)
CYP38	At3g01480	PPIase	Assembly of PSII	Sec	R	Yes	–	Fu et al. (2007), Sirpio et al. (2008), Vasudevan et al. (2012)
FKBP20-2	At3g60370	PPIase	Assembly of PSII	Tat	R	Yes	–	Lima et al. (2006)
PPL1	At3g55330	OEC, PsbP-like	Assembly of OEC	Tat	R	–	–	Ishihara et al. (2007)
CtpA	At5g46390	Peptidase	Processing of D1	Sec	R	Yes	–	Anbudurai et al. (1994)
CtpA	At4g17740	Peptidase	Processing of D1	Sec	R	Yes	–	Anbudurai et al. (1994)
CtpA	At3g57680	Peptidase	Processing of D1	No	–	–	–	Yin et al. (2008)
Deg1	At3g27925	Protease	Degradation of D1	Sec	R	Yes	–	Kapri-Pardes et al. (2007), Sun et al. (2007), Kley et al. (2011)
Deg5	At4g18370	Protease	Degradation of D1	Tat	–	Yes	–	Sun et al. (2007)
Deg8	At5g39830	Protease	Degradation of D1	Tat	R	–	–	Sun et al. (2007)
**PSI**								
PsaN	At5g64040	PSI	Subunit of PSI	Tat	C	Yes	Yes	Haldrup et al. (1999)
PPD1	At4g15510	OEC, PsbP-like	Assembly of PSI	Tat	R	–	–	Liu et al. (2012)
**Cyt b6f**								
FKBP13	At5g45680	PPIase	Assembly of cyt b6f	Tat	R	Yes	–	Gupta et al. (2002a), Gollan et al. (2011)
**NDH**								
PNSL1/PPL2	At2g39470	OEC, PsbP-like	Subunit of NDH	Tat	R	–	–	Ishihara et al. (2007)
PNSL2	At1g14150	OEC, PsbQ-like	Subunit of NDH	Tat	R	–	–	Suorsa et al. (2010), Yabuta et al. (2010)
PNSL3	At3g01440	OEC, PsbQ-like	Subunit of NDH	Tat	R	–	–	Suorsa et al. (2010), Yabuta et al. (2010)
PNSL4/FKBP16-2	At4g39710	PPIase	Subunit of NDH	Tat	R	–	–	Peng et al. (2009)
PNSL5/CYP20-2	At5g13120	PPIase	Subunit of NDH	Sec	–	–	–	Romano et al. (2004a), Shapiguzov et al. (2006), Peng et al. (2009), Sirpio et al. (2009)
PQL3	At2g01918	OEC, PsbQ-like	Subunit of NDH	Tat	–	–	–	Yabuta et al. (2010)
**Photoprotection**								
VDE	At1g08550	VDE, lipocalin	Xanthophyll cycle enzyme	Sec	–	Yes	–	Niyogi et al. (1998), Arnoux et al. (2009)
CHL	At3g47860	Lipocalin	Prevent lipid peroxidation	Sec	–	–	–	Levesque-Tremblay et al. (2009)
**Others**								
PETE2	At1g20340	Plastocyanin	Electron transport	Sec	C	–	–	Weigel et al. (2003), Pesaresi et al. (2009b)
PETE1	At1g76100	Plastocyanin	Electron transport	Sec	C	–	–	Weigel et al. (2003), Pesaresi et al. (2009b)
PRXQ	At3g26060	PRXQ-like	Antioxidant? Signaling?	No	–	Yes	–	Petersson et al. (2006)
PPD5	At5g11450	OEC, PsbP-like	Strigolactone biosynthesis	Tat	R	–	–	Roose et al. (2011)
Cyt *c*_6A_	At5g45040	Dithio-cytochrome	Oxidizing lumenal proteins	Sec	–	–	–	Gupta et al. (2002b), Weigel et al. (2003), Pesaresi et al. (2009b)
CS26	At3g03630	Cysteine synthase	*S*-sulfocysteine synthase	No	–	–	–	Bermudez et al. (2010), Bermudez et al. (2012)

*Thylakoid lumen proteome in Arabidopsis comprises of about 80 proteins of which half has a functionally verified role. Network based on Ifuku et al. (2010). R, regulatory protein; C, constitutively expressed protein. TRX target proteins based on Lindahl and Kieselbach (2009) and Hall et al. (2010).*

## DISTINCTIVE FEATURES OF THYLAKOID LUMEN

A decade ago, the thylakoid lumen was believed to be largely devoid of proteins, containing only the OEC proteins, the electron carrier plastocyanin (PC) and violaxanthin de-epoxidase (VDE). Proteomic and genomic studies have now revealed up to 80 proteins in *Arabidopsis thaliana* (*Arabidopsis*) to be localized in this compartment (Peltier et al., 2002; Schubert et al., 2002; Kieselbach and Schroder, 2003). All characterized lumenal proteins in *Arabidopsis* (**Table 1**) are nuclear-encoded and post-translationally transported into the chloroplast by the TOC/TIC (translocon at the outer/inner envelope of chloroplasts) system (Soll and Schleiff, 2004), while the secretory (Sec) and twin-arginine translocation (Tat) pathways import proteins into the lumen (discussed below; Albiniak et al., 2012). The thylakoid lumen is a constricted and crowded environment in which protein mobility is largely restricted; however, the dimensions of the thylakoid lumen are quite flexible (Mullineaux, 2008). Expansion of the lumenal space occurring in high light is linked to light-induced decrease in pH from around 7.0 in darkness to 5.8 and 6.5 in the light (Kramer et al., 1999; Cruz et al., 2001; Tikhonov, 2013) due the concomitant influx of anions upon acidification (Kirchhoff et al., 2011). The increase in lumenal space under light conditions is thought to allow protein diffusion that is important for PSII maintenance during photosynthetic activity (Kirchhoff et al., 2011). The lumenal pH controls the activities of many lumenal proteins, effectively functioning as a light-sensing on/off switch (discussed below).

## PHOTOSYNTHETIC ELECTRON TRANSFER FROM A LUMENAL PERSPECTIVE

### WATER-SPLITTING

The OEC contains a Mn_4_O_5_Ca cluster that operates in water oxidation at the lumenal side of PSII. After storing four positive charges as a result of four successive electron transfer steps, the OEC oxidizes two water molecules and releases one oxygen molecule and four protons to the thylakoid lumen. Hence the OEC both liberates electrons for the electron transport chain and participates in acidification of the lumen. The OEC is supported by an extrinsic lumen protein complex, which reversibly associates with intrinsic PSII proteins. The OEC proteins are PsbO (also called OEC33), which is located proximal to the Mn_4_O_5_Ca cluster, PsbP (OEC23) and PsbQ (OEC17; Bricker et al., 2012). In *Arabidopsis* each OEC protein is encoded by two duplicate genes. The PsbO1 isoform exhibits higher oxygen-evolving activity than PsbO2 and accounts for around 90% of the total PsbO in WT plants (Murakami et al., 2005). PsbQ1 differs from PsbQ2 in a phosphorylatable serine residue that occurs in the latter (Reiland et al., 2009), while the PsbP1 is the major isoform of PsbP in the Columbia-0 ecotype since the *PSBP2* gene contains a frameshift that leads to truncated PsbP2 protein that is probably excluded from the thylakoids (Ifuku et al., 2008).

All three OEC proteins are required for maximal oxygen evolution, most likely because they sequester Cl^-^ and Ca^2^^+^ ions required for water-splitting (Miqyass et al., 2007; Popelkova and Yocum, 2007). Additionally, each of the OEC proteins appears to have a unique role in the integrity of PSII complexes. PsbQ is important for PSII stability, particularly under low light (Yi et al., 2006), while PsbP is required for assembly and/or stability of PSII and formation of PSII–LHCII supercomplexes (Yi et al., 2007; Ido et al., 2009). PsbQ stabilizes the interaction between PsbP and the membrane-bound PSII subunit PsbR (Suorsa et al., 2006; Allahverdiyeva et al., 2013). These results suggest that PsbP and PsbQ may coordinate the removal and/or reintegration of the Mn_4_O_5_Ca cluster with the disassembly and/or reassembly of PSII complexes during the PSII repair cycle (De Las Rivas et al., 2007). In addition, PsbP and PsbQ are linked to granal stacking (Dekker and Boekema, 2005), but evidence regarding the specific role of PsbQ in thylakoid architecture is contradictory (Yi et al., 2009). PsbO has been described as a GTPase that regulates PSII repair (Spetea et al., 2004; Lundin et al., 2007) and as a carbonic anhydrase (Lu et al., 2005), and has also demonstrated Ca^2^^+^ ion-binding activity (Heredia and De Las Rivas, 2003; Murray and Barber, 2006), although all of these features of PsbO are somewhat contentious and remain to be unequivocally demonstrated.

### THE Q CYCLE AND cyt *b_6_*f

Part of electrons from PSII is shuttled to cyt *b_6_*f via the so-called “Q cycle,” which involves successive reduction and oxidation of the membrane-soluble electron- and proton-carrier plastoquinone (PQ). Each Q cycle pumps two protons from the stroma to the lumen, coupling the pH of the lumen to PET activity (Tikhonov, 2013). A subunit of the cyt *b_6_*f complex known as the Rieske protein has a lumenal [2Fe-2S] cluster-binding domain that operates in electron transfer between cyt *b*_6_ and cyt *f*. Rieske interacts with the lumenal immunophilin FKBP13 (Gupta et al., 2002a; Gollan et al., 2011), which was thought to regulate the assembly of the cyt *b_6_*f complex from the stromal side (Gupta et al., 2002a), although recent results suggest that the FKBP13–Rieske interaction occurs in the thylakoid lumen (Gollan et al., 2011). The chaperone activity of FKBP13 is sensitive to redox regulation, as discussed below.

### PLASTOCYANIN AND PSI

Electron transfer from cyt *b_6_*f to PSI takes place in the lumen, and yet few lumen proteins appear to be directly involved. The major lumenal electron carrier is the copper-containing protein PC, comprising two isoforms in *Arabidopsis* that are encoded by the *PETE1* and *PETE2* genes, of which the latter is more highly expressed (Pesaresi et al., 2009b). A cyt *c* protein that operates as an alternative electron donor for PSI in cyanobacteria and green algae also occurs in the *Arabidopsis* thylakoid lumen (cyt *c*_6__A_), where it has been suggested to have a similar function (Gupta et al., 2002b); however, strong evidence suggests this is not the case in *Arabidopsis* (Weigel et al., 2003; Pesaresi et al., 2009b). PsaN is the only lumenal subunit of PSI (Kieselbach and Schroder, 2003). Mutants lacking PsaN are capable of assembling functional PSI complexes and growing photoautotrophically; however, restricted electron flow between PSII and PSI in mutant plants show that PsaN is necessary for efficient interaction between PSI and PC (Haldrup et al., 1999).

### CYCLIC ELECTRON TRANSFER

In PSI CET, electrons are directed from ferredoxin (Fd) back into the Q cycle rather than to NADP^+^. The commonly accepted role of CET is in adjusting stromal ATP:NADPH ratios in response to metabolic requirements; however, CET also operates to maintain the low lumenal pH required for NPQ and photosynthetic control of cyt *b_6_*f to protect both PSII and PSI, particularly under conditions where PSII is disengaged or inhibited (Kramer et al., 2004; Munekage et al., 2004; Joliot and Johnson, 2011). PSI CET proceeds by two partially redundant pathways; the major route is dependent on proton gradient regulation (PGR) proteins (Munekage et al., 2002; Okegawa et al., 2007) and the formation of a multi-protein CET supercomplex (DalCorso et al., 2008; Iwai et al., 2010), although some of the components remain to be discovered.

The second, minor route for PSI CET involves the membrane-intrinsic NADPH dehydrogenase-like (NDH) complex, which also forms a CET-specific supercomplex through association with PSI (Rumeau et al., 2007; Peng et al., 2009). Based on the structural similarities with mitochondrial complex I of the respiratory chain (Friedrich and Weiss, 1997), which oxidizes NADH and reduces ubiquinone in a process that is coupled to proton translocation across the mitochondrial inner membrane, the NDH-like complex is proposed to play a similar role in the thylakoid membrane. However, the physiological relevance, functional mechanism, and regulation of the chloroplast NDH-like complex have not been fully elucidated (Shikanai, 2007; Yamamoto et al., 2011), partly due to the fact that the abundance of NDH-like complexes in the thylakoid membrane is very low (Sazanov et al., 1998). Nevertheless, the complex is known to be stable only when associated with at least two copies of PSI, and the role of NDH in PSI CET and chlororespiration has been established (Peng and Shikanai, 2011).

The NDH-like complex is composed of at least 30 subunits and auxiliary proteins (Ifuku et al., 2011), and thus the PSI–NDH supercomplex is among the largest protein complexes in the thylakoid membrane. The subunits of NDH include both nuclear-encoded and plastid-encoded proteins, indicating strict control of expression, protein import and assembly processes. Based on characterization of *ndh* mutant *Arabidopsis* lines, the chloroplast NDH is postulated to comprise four subcomplexes, known as “A,” “B,” “membrane,” and “lumen” subcomplexes (Ifuku et al., 2011), although detailed structural data for any of these is currently missing. The higher plant NDH is closely related to its cyanobacterial counterpart, with the major differences being the lumenal subcomplex and some auxiliary proteins that are characteristic to plant NDH (Peng et al., 2009; Battchikova et al., 2011).

The lumenal subcomplex, which is vital for stability of subcomplex A, comprises a PsbP homolog (PPL2, also called PNSL1), a PsbQ homolog (PNSL2), and immunophilins FKBP16-2 (PNSL4) and CYP20-2 (PNSL5; Peng et al., 2009; Sirpio et al., 2009; Suorsa et al., 2010; Yabuta et al., 2010). Of these PNSL5/CYP20-2 is the sole contributor to cyclophilins (CYP)-mediated lumenal PPIase activity (Shapiguzov et al., 2006) and was initially found to co-migrate with LHCII (Romano et al., 2004a). Incorporation of subcomplex A into the thylakoid membrane is one of the final steps in formation of functional NDH, and it may be a reversible one that can disengage CET or accommodate NDH repair (Peng and Shikanai, 2011). It seems plausible that the lumenal subcomplex could regulate the assembly and/or (more likely) disassembly of NDH according to the conditions in the thylakoid lumen.

## THE PSII ASSEMBLY AND REPAIR INVOLVE A LARGE ARRAY OF LUMENAL PROTEINS

Photosystem II biogenesis shares many components with the repair cycle occurring after photoinhibition of PSII. The D1 protein in the PSII reaction center is the major target of irreversible photodamage during photosynthesis under high light, leading to NPQ by photoinhibition-related quenching (qI); however, balanced damage and repair of PSII have been shown to occur at all light intensities (Tyystjärvi and Aro, 1996). Replacement of damaged D1 requires disassembly of PSII–LHCII complexes, PSII migration from crowded grana thylakoids to the stromal lamellae, D1 removal and replacement, reassembly and finally relocation of functional PSII (Baena-González and Aro, 2002). The lumenal components of PSII biogenesis/repair cycle are discussed below.

Degradation of the damaged D1 protein, carried out primarily by thylakoid-associated FtsH proteases, occurs in cooperation with the lumenal Deg1, Deg5, and Deg8 proteases (Kato et al., 2012). Deg1 is activated by homo-hexamerization in response to pH changes in the lumen (Kley et al., 2011), and interaction between Deg5 and Deg8 to form an active protease complex may also be pH-dependent (Sun et al., 2007). While activated, Deg proteases specifically degrade lumen-exposed loops of D1 (Kapri-Pardes et al., 2007; Sun et al., 2007). Deg1 has proteolytic activity against other lumenal proteins *in vitro*, including PC and PsbO, suggesting it may operate as a general protease in the thylakoid lumen (Chassin et al., 2002). In addition to proteolytic activity, Deg1 assists PSII assembly through interaction with the reaction center protein D2 (Sun et al., 2010b). Interestingly, the thylakoid lumen acidic phosphatase TLP18.3 is also involved in the degradation of D1 protein, but also in dimerization of PSII (Sirpio et al., 2007; Wu et al., 2011). Interaction between TLP18.3 and Deg1 (Zienkiewicz et al., 2012) might regulate the protease through dephosphorylation (Spetea and Lundin, 2012). The D1 protein is the primary target of photodamage, but other PSII core proteins are also damaged and degraded, particularly in response to environmental stresses. Stromal Deg7 has been shown to be involved in the proteolysis of photodamaged D1, D2, CP47, and CP43 (Sun et al., 2010a), while stromal FtsH proteases and lumenal Deg1 mediate the degradation of LHCII proteins (Zienkiewicz et al., 2012; Luciński and Jackowski, 2013).

Newly synthesized D1 protein is co-translationally inserted into the thylakoid membrane and the core complex. The latter step is assisted by two lumenal proteins, immunophilin CYP38 and “high chlorophyll fluorescence 136” (HCF136), both of which are present already in the proteome of pre-chloroplastic etioplasts, presumably for prompt D1 assembly during thylakoid biogenesis (Meurer et al., 1998; Kanervo et al., 2008). The HCF136 protein is a prerequisite for the assembly of PSII reaction centers during complex biogenesis, while CYP38 assists the assembly of PSII core complexes during both biogenesis and repair (Meurer et al., 1998; Plucken et al., 2002; Fu et al., 2007; Sirpio et al., 2008). The C-terminal CYP-like domain of CYP38 interacts with the PSII apoprotein, CP47 (Vasudevan et al., 2012). Thus, CYP38 might assist the correct folding and integration of CP47 into the PSII core. An additional role for CYP38 may lie in the regulation of correct conformation of D1 and possibly also CP43 during PSII biogenesis and/or repair and as a negative regulator of the thylakoid protein phosphatase that dephosphorylates PSII core proteins (Fulgosi et al., 1998; Vener et al., 1999; Rokka et al., 2000; Fu et al., 2007; Sirpio et al., 2008). The lumenal immunophilin FKBP20-2 also has a role in PSII complex assembly by a yet unknown mechanism (Lima et al., 2006).

Processing of precursor D1 protein to the mature form by the C-terminal processing protease CtpA (Anbudurai et al., 1994; Yamamoto et al., 2001) is required for integration of the OEC complex to PSII (Roose and Pakrasi, 2004). The lumen proteome of *Arabidopsis* includes three CtpA homologs. Mutation in one of these genes (At3g57680) does not affect accumulation of the D1 precursor suggesting that there may be functional redundancy between the CtpA homologs (Yin et al., 2008). The lumenal homologs Psb27 and “low PSII accumulation 19” (LPA19) interact with the newly inserted D1 precursor and are involved in processing of nascent D1 during PSII biogenesis in *Arabidopsis* (Chen et al., 2006; Wei et al., 2010). The Psb27 homolog in cyanobacteria interacts with PSII to prevent premature assembly of the Mn_4_O_5_Ca cluster at the lumenal side of PSII (Roose and Pakrasi, 2008), suggesting that the timing of D1 maturation is important in the PSII assembly. The importance of a PsbP homolog PPL1 for the PSII repair cycle was shown by the slow recovery of PSII from photoinhibition in *ppl1* plants (Ishihara et al., 2007). Finally, the lumenal ascorbate peroxidase APX4/TL29 has been described as a lumen-located component and/or auxiliary protein of PSII (Granlund et al., 2009b), although according to its crystal structure its function is unlikely to involve peroxidase activity (Lundberg et al., 2011).

## PSI ASSEMBLY IS DEPENDENT ON LUMENAL PsbP-LIKE PROTEIN PPD1

Compared to PSII, PSI is much more tolerant to, and/or very well protected from photoinhibition, as PSI photodamage exists *in vivo* only under specific conditions such as chilling temperature (Zhang and Scheller, 2004) or in the deficiency of PGR-dependent CET (Suorsa et al., 2012a, 2012b). So far only one lumenal protein assisting PSI biogenesis, namely the PsbP-like protein PPD1, has been identified. PPD1 interacts directly with PSI reaction center proteins PsaA and PsaB and assists the folding and insertion of these two proteins into the thylakoid membrane (Liu et al., 2012). A lack of PPD1 leads to the loss of PSI and an inability to grow photoautotrophically (Liu et al., 2012).

## PHOTOPROTECTION COMPONENTS IN THE THYLAKOID LUMEN

In naturally fluctuating light conditions, the energy harvested by LHCII can become unbalanced in relation to the capacity of stromal acceptors, thus saturating the electron transport chain and generating reactive oxygen species (ROS) that cause photodamage of membrane proteins (Nishiyama et al., 2006; Murata et al., 2007). In order to protect the photosynthetic machinery amidst natural light conditions, plants use energy dissipation mechanisms (NPQ) that are partially located in the thylakoid lumen (Niyogi et al., 1998).

### NON-PHOTOCHEMICAL QUENCHING

The major NPQ mechanism (qE) is rapid and reversible, involving dissipation of absorbed light energy as heat. This is predominantly achieved through production of the carotenoid zeaxanthin and reorganization of LHCII, both processes that are triggered by acidification of the thylakoid lumen. Upon protonation, lumenal VDE converts from a monomer to a dimer, opening access to the active site that facilitates the conversion of violaxanthin to zeaxanthin (Arnoux et al., 2009). Protonation of the PSII protein PsbS causes a structural rearrangement of PSII–LHCII supercomplexes (Li et al., 2000, 2004; Kereïche et al., 2010), although the exact role of PsbS in qE remains to be defined (Johnson and Ruban, 2011).

### LUMEN RESPONSE TO OXIDATIVE STRESS

Cysteine synthesis 26 (CS26) is an *S*-sulfocysteine synthase and occurs in low abundance in the thylakoid lumen, but it has a vital role in detection of lumenal redox conditions, particularly in long photoperiods (Pesaresi et al., 2009a; Bermudez et al., 2010, 2012). A lack of CS26 led to strong photoinhibition and a systemic ROS response that was accompanied by reduced levels of OEC proteins and PSII assembly factors (Bermudez et al., 2012). CS26 was recently proposed as a ROS sensor through its sensitivity to thiosulfate accumulation in the lumen (Gotor and Romero, 2013). The “chloroplastic lipocalin” (CHL) is involved in photoprotection of thylakoid membrane lipids. CHL accumulates in the thylakoid lumen during environmental stress conditions such as drought and high light, as well as in paraquat and abscisic acid treatments, to protect the thylakoid membrane from peroxidation (Levesque-Tremblay et al., 2009).

## LUMEN PROTEIN FAMILIES

### DIVERSE ROLES OF THE PsbP-LIKE AND PsbQ-LIKE PROTEINS

The PsbP family has at least ten members in the *Arabidopsis* thylakoid lumen (Hall et al., 2010; Sato, 2010). Aside from the OEC protein PsbP, these are PPL1 and PPL2, involved in PSII repair and NDH stability, respectively (discussed above), and at least seven “PsbP domain” proteins (PPD1–7). An eighth (PPD8) is encoded, but has not been detected at the protein level. The role of PPD1 in PSI assembly has been discussed above, but the specific activities of other PPDs in the lumen remain a mystery in many respects. A homolog of PPD2 in the green alga *Chlamydomonas reinhardtii* is implicated in the generation of singlet oxygen signals (Brzezowski et al., 2012) and PPD5 knockout in *Arabidopsis* led to a reduction in NDH activity and is linked to production of the carotenoid-derived hormone strigolactone (Roose et al., 2011).

Similarly, multiple PsbQ-like proteins occur in the *Arabidopsis* lumen. PQL1 and PQL2 are lumenal subunits of NDH (see above), while a third (PQL3) is also required for NDH function, but has not been found in the proteome (Yabuta et al., 2010). The cyanobacterial ancestors of plant PsbP and PsbQ domains, called “cyanoP” and “cyanoQ,” respectively, are involved in PSII oxygen evolution, but may have more of an auxiliary role in regulation of OEC structure and assembly. Notably, cyanoP is considerably more closely related, at least in sequence and structure, to PPL1 than to PsbP in plants (Sato, 2010; Jackson et al., 2012). Considering the few details about the PsbP- and PsbQ-like proteins known so far, it is tempting to speculate that expansion of these families in the lumen has provided opportunities for regulating the lumen-exposed parts of various photosynthetic complexes.

### LUMENAL IMMUNOPHILINS REGULATE THE ASSEMBLY, MAINTENANCE, AND TURNOVER OF THYLAKOID MEMBRANE PROTEIN COMPLEXES

The immunophilins include two unrelated protein families, the CYP and the FK506-binding proteins (FKBP), both of which are abundant in the thylakoid lumen proteome (He et al., 2004). Immunophilins are well known for their ability to rotate the peptide bond of a proline residue, known as PPIase activity, which has been linked to protein folding; however, a majority of the lumenal immunophilins does not show PPIase activity against synthetic peptides (Shapiguzov et al., 2006; Edvardsson et al., 2007). The best characterized of the lumen immunophilins is CYP38, which has an atypical CYP domain in the C-terminus and an N-terminal helical bundle, possibly for autoinhibition (Vasudevan et al., 2012). CYP38 does not show PPIase activity, but has a vital role in the assembly of PSII (Fu et al., 2007; Sirpio et al., 2008). Contrary to earlier observations (He et al., 2004; Romano et al., 2004b; Sirpio et al., 2008), CYP38 in *Arabidopsis* lacks a leucine zipper domain due to a frameshift in the coding sequence. The spinach ortholog of CYP38, called “thylakoid lumen PPIase of 40 kDa” (TLP40; 82% sequence identity to CYP38) is likely to possess a similar functional role to CYP38, but appears to behave differently to its *Arabidopsis* counterpart in that TLP40 has PPIase activity *in vitro* (Fulgosi et al., 1998; Vener et al., 1999). FKBP20-2 was also implicated in PSII assembly based on the observed increase of unassembled PSII monomers and dimers in the *fkbp20-2* knockout, suggesting a role in formation of PSII supercomplexes (Lima et al., 2006). As discussed earlier, FKBP16-2 and CYP20-2 take part in the lumenal NDH subcomplex (Peng et al., 2009), while another immunophilin, FKBP13, is linked to cyt *b_6_*f regulation through interaction with Rieske (Gupta et al., 2002a; Gollan et al., 2011). In wheat, FKBP16-1 and FKBP16-3 may have a role in development of photosynthetic membranes through their interaction partners, the PsaL subunit of PSI and “thylakoid formation-1” (THF1, also called PSB29), respectively (Gollan et al., 2011).

The roles of most lumenal immunophilins remain unclear, although accumulating evidence indicates a primary role in the assembly and/or turnover of photosynthetic complexes. FKBP16-2, FKBP16-4, and CYP37 have been found both in the membrane-bound and lumen-soluble thylakoid proteomes (Peltier et al., 2002; Friso et al., 2004), suggesting that they may be involved in recruitment of lumen proteins to the membrane.

### PENTAPEPTIDE REPEAT PROTEINS IN THYLAKOID LUMEN HAVE UNKNOWN FUNCTION

A lumenal pentapeptide repeat-containing (PPR) family has three members; TL15, TL17, and TL20.3 (Schubert et al., 2002; Hall et al., 2010). The lumenal pentapeptide proteins TL15 and TL17 in *Arabidopsis* increase in abundance upon light adaptation (Granlund et al., 2009a) and are, together with TL20.3, putative targets of thioredoxin (TRX) reduction (Hall et al., 2010). In line with this, the crystal structure of TL15 has revealed an internal disulfide bridge (Ni et al., 2011). Cyanobacterial PPRs have diverse roles, two of which may be relevant in the thylakoid lumen; regulation of light-induced manganese ion import (Chandler et al., 2003) and galactolipid translocation (Black et al., 1995).

## POST-TRANSLATIONAL MODIFICATIONS OF LUMEN PROTEINS

### REGULATION OF LUMEN PROTEINS BY REVERSIBLE PHOSPHORYLATION

Phosphoproteomics studies have identified several phosphorylated proteins in the thylakoid lumen (**Table 1**), including the OEC proteins PsbP and PsbQ (Reiland et al., 2009) and lumen-exposed regions of the PSII subunits PsbR and CP47 (Reiland et al., 2009) and the PSI subunits PsaF (Sugiyama et al., 2008) and PsaN (Stael et al., 2012). Phosphorylation of photosynthetic proteins is thought to regulate assembly of the photosynthetic machinery in response to environmental conditions (Reiland et al., 2009). The recent discovery that PsaN phosphorylation is calcium-dependent may link PSI maintenance with dark-induced stromal Ca^2^^+^ flux (Stael et al., 2012). Despite these results, neither lumenal kinases, nor the physiological significance of phosphorylation events in the lumen have been found, while a single candidate for dephosphorylation activity is the membrane anchored TLP18.3 (Sirpio et al., 2007; Wu et al., 2011), although its substrates are unknown. The existence of any nucleotide-dependent processes in the lumen is contentious (Kieselbach and Schroder, 2003), although accumulating evidence suggests that ATP can be imported to the lumen by a membrane-embedded thylakoid ADP/ATP carrier (TAAC; Thuswaldner et al., 2007), where it is presumed to be available for protein phosphorylation. Recently TAAC was also described as a phosphosulfate channel in the plastid envelope (Gigolashvili et al., 2012). A nucleoside diphosphate kinase 3 (NDK3) found both in the thylakoid lumen and in mitochondria is capable of hydrolyzing ATP to generate GTP thought to be the substrate for GTPase activity of PsbO that is implicated in OEC dissociation for PSII repair cycle (Spetea and Lundin, 2012).

### REDOX REGULATION THROUGH DISULFIDE BRIDGE MODULATION

According to current knowledge, more than 40% of the lumen proteome may be regulated by redox reactions through modulation of disulfide bonds that control protein translocation and folding and/or enzyme activation (Hall et al., 2010). This observation places lumenal redox enzymes as powerful regulators of numerous processes. In comparison, less than 10% of stromal proteins are regulated by TRX, although at least 10 TRX isoforms exist in the stroma. Chloroplast redox enzymes have recently been thoroughly reviewed (Lindahl and Kieselbach, 2009; Hall et al., 2010), and will be discussed here only briefly.

The leading candidate for the source of disulfide reduction in the lumen is HCF164, an integral membrane enzyme with a lumenal TRX domain, thought to accept reducing equivalents from stromal TRX via the membrane-localized “cyt *c* defective A” (CcdA; Motohashi and Hisabori, 2006, 2010). HCF164 interacts with cyt *f* and the Rieske iron–sulfur protein and is required for assembly of the cyt *b_6_*f complex (Lennartz et al., 2001), and is also capable of reducing PsaN (Motohashi and Hisabori, 2006). A similarly membrane-embedded TRX-like protein is the “suppressor of quenching 1” (SOQ1), thought to regulate NPQ through a previously uncharacterized pathway (Brooks et al., 2013). “Low quantum yield of photosystem II” (LQY1) is a thylakoid membrane-bound Zn finger protein with protein disulfide isomerase activity that interacts with PSII core complexes to modulate disulfide bond formation in PSII subunits during the PSII repair cycle (Lu et al., 2011). “Peroxiredoxin Q” (PRXQ) generally transfers reductants from TRX to hydrogen peroxide for detoxification; however, lumenal PRXQ does not appear to reduce hydrogen peroxide (Petersson et al., 2006).

Disulfide bond formation in the lumen requires an electron acceptor to oxidize thiol groups, although the mechanism for this is not clear. One prospect is lumenal oxygen that is released by water-splitting reactions (Buchanan and Luan, 2005). In an interesting development of this idea, CS26 was proposed to regulate thiol oxidation by production of *S*-sulfocysteine in the lumen (Bermudez et al., 2012). Another candidate thiol oxidase is the lumenal cyt *c*_6A_, which is proposed to shuttle reducing equivalents between thiols and PC (Schlarb-Ridley et al., 2006). Recently the “lumen thiol oxidoreductase 1” (LTO1) protein was found to be a thylakoid membrane-localized enzyme with a lumenal TRX domain that was recently shown to catalyze disulfide bond formation in PsbO *in vitro* (Karamoko et al., 2011).

Although the mechanisms of thiol/disulfide modulation in the lumen remain unclear, important photosynthetic processes are redox-regulated. Disulfide bond formation is important for folding of PsbO1 and PsbO2, which are susceptible to proteolysis in their unfolded state (Hashimoto et al., 1997; Hall et al., 2010; Karamoko et al., 2011). VDE contains disulfides that are vital for its activity in NPQ (Hall et al., 2010). The substrate-binding/PPIase activity of FKBP13 is controlled by two disulfide bridges that can be reduced and oxidized *in vitro* by TRX (Gopalan et al., 2004, 2006) and LTO1 (Lu et al., 2013), respectively. This suggests that the interaction between FKBP13 and the Rieske iron–sulfur protein may be linked to redox state of the thylakoid (Gollan et al., 2011). Furthermore, homology between FKBP13 and FKBP16-2 infers similar redox sensitivity for the assembly of the lumenal NDH subcomplex (Gollan et al., 2012), although these possibilities have not been tested experimentally. The activity of lumen immunophilins FKBP20-2 and CYP38 may also be regulated by disulfide bond modulation (Lima et al., 2006; Fu et al., 2007; Sirpio et al., 2008). Identification of lumen TRX targets indicates that the PSII repair cycle and OEC assembly are under redox control (Hall et al., 2010). Finally, a lumen-exposed disulfide bridge is thought to regulate the activity of the membrane-bound LHCII kinase STN7 (Lemeille et al., 2009), although the redox factors responsible have not been found.

## PROTEIN TRANSLOCATION INTO THYLAKOID LUMEN

Four separate methods of protein import into thylakoids are established; the “signal recognition particle-dependent” (SRP) method and the “spontaneous” method insert integral membrane proteins into the thylakoid membrane and are employed by many photosynthetic subunits (Michl et al., 1994; Kim et al., 1999). Lumen proteins are translocated from the chloroplast stroma by either the Sec pathway or the Tat pathway, depending on the signal peptide in the precursor of the passenger protein (Albiniak et al., 2012; **Table 1**).

The Sec system comprises three components; SecA binds the signal peptide in the passenger protein, hydrolyses ATP and threads the unfolded precursor through a fixed channel in the thylakoid membrane comprising SecE and SecY subunits (Yuan et al., 1994; Laidler et al., 1995; Schuenemann et al., 1999). Sec substrates include PsbO, PC, and VDE (Mori et al., 1999).

Unlike the Sec pathway, the Tat pathway operates independently of ATP hydrolysis, instead deriving energy from the *pmf* across the thylakoid membrane. The Tat system comprises three integral membrane subunits; “high chlorophyll fluorescence 106” (Hcf106) and cpTatC associate together to form a large, hetero-oligomeric complex in the thylakoid membrane, while Tha4 occurs in separate homo-oligomeric complexes. The signal peptides of Tat passengers conserve a central, basic “Arg-Arg” motif that is recognized by the Hcf106–cpTatC receptor complex which, in the presence of suitable *pmf*, then transiently associates with Tha4, which, according to the current model, forms the translocation pore to conduct the passenger protein through a membrane (Albiniak et al., 2012). According to their signal peptides, all PsbP and PsbQ proteins and their homologs in *Arabidopsis* are Tat substrates, as are all lumenal FKBPs (including FKBP16-2; Gollan et al., 2012). A compelling feature of the Tat pathway is its capacity to transport folded proteins and protein–cofactor complexes. In the homologous bacterial Tat system, this is a “quality control” mechanism that ensures proper protein folding and cofactor integration prior to protein export (Hynds et al., 1998; Berks et al., 2000). In plants the Tat pathway could similarly facilitate folding and assembly in the relatively stable environment of the chloroplast stroma to underwrite protein and cofactor integrity in the fluctuating conditions of the lumen (Muller and Klosgen, 2005). Furthermore, thylakoid import of folded proteins could abrogate the need for post-translational modifications such as phosphorylation in the lumen. It should be noted that important details of the Tat pathway in plants remain unclear, including (i) the physical mechanism of translocation, (ii) contributions of the *pmf* components, (iii) involvement of stromal chaperones, and (iv) the conformations, post-translational modifications and complex states of Tat passengers.

## RESPONSE OF THE LUMEN PROTEOME TO ENVIRONMENTAL CUES

### TRANSCRIPTION REGULATION

The importance of retrograde signals emitted from the chloroplast, and from other sites in the plant cell, in regulating the nuclear expression of photosynthetic proteins is becoming clear (Foyer et al., 2012; Queval and Foyer, 2012). Similar signaling factors are likely to regulate expression of lumenal proteins, which are all encoded in the nucleus (**Table 1**), and yet elucidation of these signals has received little attention. A recent analysis of the expression profiles divided lumen proteins into two networks; a “constitutive factor” group that included predominantly PSI and PSII subunits and few PSII auxiliary proteins, and a “regulatory factor” group containing NDH subunits, as well as several proteins involved in PSII regulation (Ifuku et al., 2010).

### ACCLIMATIONS OF THE LUMEN PROTEOME TO LIGHT AND TEMPERATURE

Fifteen thylakoid lumen proteins displayed increased abundance in light-adapted *Arabidopsis* compared to dark-adapted plants indicating that their roles are related to photosynthetic activity (Granlund et al., 2009a). These include OEC proteins PsbP1 and PsbQ2, PSII auxiliary proteins HCF136 and PPL1 as well as major PC (PETE2). Additionally PPD5, two pentapeptide proteins and a group of other functionally uncharacterized thylakoid lumen proteins are up-regulated at the protein level in light compared to darkness (Granlund et al., 2009a). Notably, a majority of the proteins found in higher abundance in the light-adapted lumen are Tat substrates, suggesting that the Tat system may regulate the lumen proteome in response to prevailing light (and other stress) conditions according to the *pmf* that is generated.

Acclimation to low temperature affects the accumulation of eight thylakoid lumen proteins in *Arabidopsis* (Goulas et al., 2006). These include PsbO1/2, PsbP1/2 proteins, HCF136, NDH related immunophilin PNSL5/CYP20-2, and two FKBP proteins. The drastic increase in accumulation of PNSL5/CYP20-2, which occurs concomitantly with down-regulation of the Calvin–Benson cycle enzymes during cold acclimation, might be linked to the activation of NDH-dependent CET under such conditions. However, it should be noted that *Arabidopsis* is a cold-tolerant plant and a different response, e.g., in the accumulation of the NDH-like complex, could be present in rice or other cold-sensitive plant species.

### THE IMPORTANCE OF PH AS A REGULATOR OF LUMEN PROTEIN ACTIVITY

**FIGURE 2:**
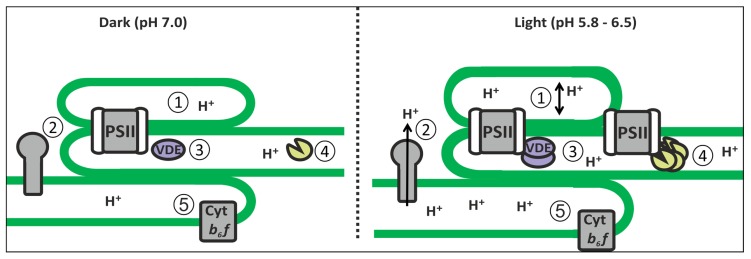
**Lumenal processes under pH regulation.** (1) Light-induced expansion of the lumen volume facilitating plastocyanin migration and (2) enhancement of ATP synthase activity; (3) Light-induced activation of VDE and PsbS by protonation for photoprotection; (4) Deg oligomerization for PSII repair; and (5) induction of photosynthetic control via cyt *b*_*6*_f.

Light-induced protonation of the thylakoid lumen contributes the major portion of the *pmf* that drives ATP production; however, the acidic lumen is an important factor in many other processes, as reviewed above (**Figure 2**). Low pH is required to regulate electron transport, through qE activation and photosynthetic control of cyt *b_6_*f (Bratt et al., 1995; Kramer et al., 2004; Li et al., 2004; Joliot and Johnson, 2011). pH-dependent oligomerization of Deg proteases connects thylakoid lumen pH to photoinhibition, recovery and the proteolytic breakdown of other lumenal proteins (Hall et al., 2010). Likewise, OEC is known to become inactivated by pH below 6.0 (Commet et al., 2012). Finally, the light- and dark-induced changes in thylakoid membrane architecture, and the internal dimensions of the thylakoids, are also linked to thylakoid lumen pH (Kirchhoff et al., 2011). The pH of the lumen is determined by the respective rates of electron transfer and ATP synthase activity, and regulation of these processes is used to maintain stromal homeostasis (Kramer et al., 2004; Joliot and Johnson, 2011). It stands to reason that other lumenal activities may also be regulated according to metabolic requirements through controlled changes in thylakoid lumen pH.

## CONCLUDING REMARKS

The thylakoid lumen not only provides the environment for oxygen evolution, PC-mediated electron transfer and zeaxanthin formation, but also houses factors that are important for the biogenesis, maintenance and turnover of photosynthetic protein complexes, activity of the NDH-like complex and, based on recent findings, even various signaling cascades. Indeed, most characterized lumenal proteins are linked to the PSII and NDH-like complexes, while only few are associated with PSI or cyt *b_6_*f complexes and none have functions related to ATP synthase (**Figure 1**). A striking feature of the thylakoid lumen proteome is the presence of large protein families such as the OEC-like proteins and immunophilins, suggesting that neofunctionalization of lumenal protein homologs in regulation of photosynthetic complexes has driven the evolution of the lumen proteome. It is evident that lumenal proteins are imported, regulated and degraded directly by changes in the lumenal conditions that reflect the metabolic requirements of the plant. Several novel retrograde and anterograde signaling networks regulating expression and activity of lumen proteins according to environmental cues are likely to be revealed during forthcoming years. To that end, the multitude of photosynthetic regulatory proteins located in the thylakoid lumen should be carefully considered when identifying targets for improving photosynthetic reactions through genetic modifications and/or selection.

## Conflict of Interest Statement

The authors declare that the research was conducted in the absence of any commercial or financial relationships that could be construed as a potential conflict of interest.
